# Perceived Benefit, Satisfaction, Burnout, and Retention Intent Following a Structured ICU Mentorship Program for New Graduate Nurses: A Descriptive Program Evaluation

**DOI:** 10.4236/ojn.2026.1610044

**Published:** 2026-10-27

**Authors:** Margaret J. Schenk

**Affiliations:** Department of Nursing, Medical Intensive Care Unit, Critical Care Division, University of Colorado Hospital, Aurora, CO, USA

**Keywords:** Mentorship, Workforce Retention, Professional Development, Critical Care Nursing, Retention Intent, Burnout

## Abstract

**Background:**

Transitioning from nursing education to independent intensive care unit (ICU) practice can be challenging for newly graduated nurses. Nurses starting their careers within ICUs frequently experience stress, emotional exhaustion, and transition-to-practice challenges that contribute to burnout and workforce attrition. Structured mentorship may provide additional professional and emotional support during this transition.

**Methods:**

We instituted a mentorship program and subsequently conducted a post-program descriptive survey of a structured ICU mentorship program at a single academic medical center from March 2025 to May 2026. Newly graduated ICU nurses were paired with experienced ICU nurse mentors and participated in monthly mentor-mentee meetings for 6 – 12 months using a structured digital platform. Post-program electronic surveys were voluntarily completed by mentees and mentors. Outcomes were summarized descriptively.

**Results:**

We enrolled 24 participants (12 mentors and 12 mentees) in the mentorship program. Twelve mentors and 11 mentees responded to the post program survey. Among mentees, 5/11 (45%) selected a rating of 4 – 5 on a 5-point scale resulting in a high perceived benefit of mentorship; 8/11 (73%) selected a benefit rating of 3 – 5. All mentees (11/11, 100%) reported intent to continue practicing as an ICU RN after their first year. Among mentors, 11/12 (92%) reported increased satisfaction in their work as a bedside nurse, and 10/12 (83%) reported intent to continue practicing as an ICU RN for the next 1+ years. Participant perceptions following program participation were positive for both mentors and mentees with regard to burnout measures and retention outcomes.

**Conclusions:**

In this small, single-center program evaluation, participants reported perceived benefit, satisfaction, and intent to remain in ICU nursing following participation in a structured mentorship program. Expansion of the program has occurred hospital wide. These findings are positive, but warrant confirmation in larger studies using validated measures and longitudinal retention outcomes.

## 1. Introduction

The transition from nursing education to clinical practice is a critical and often challenging period, particularly for nurses entering high-acuity environments such as intensive care units (ICUs) [1] [2]. Newly graduated nurses frequently encounter increased responsibility, emotional stress, and complex patient care demands, which can contribute to burnout and early career attrition. Studies estimate that up to one-third of new nurses leave their first position within the first year, with higher rates observed in critical care settings (Table 1) [3]-[19]. Burnout among ICU nurses is well documented and is characterized by emotional exhaustion, depersonalization, and reduced personal accomplishment. These factors not only affect nurse well-being but are also associated with decreased quality of care, increased medical errors, and higher turnover rates. The COVID-19 pandemic has further exacerbated these challenges, intensifying workforce shortages and highlighting the need for sustainable retention strategies [3]-[10].

The transition from student to practicing nurse has been described as a period of transition shock, during which newly graduated nurses may experience a gap between expectations and the realities of professional practice. Mentoring can provide role modeling, professional guidance, and psychosocial support during this transition. In this program, mentorship was designed as an adjunct to orientation and nurse residency activities rather than a replacement for either process. Mentorship has emerged as a key intervention to support new graduate nurses during this transition period. Defined as a structured relationship between an experienced clinician and a less experienced individual, mentorship promotes professional development, enhances clinical confidence, and fosters resilience. Existing transition and mentorship programs vary in structure, duration, and outcomes, and less is known about structured, digitally supported mentorship delivered specifically to new graduate ICU nurses. Previous studies have demonstrated that mentorship programs can improve job satisfaction, communication skills, and retention rates; however, variability exists in program structure and outcomes, particularly within ICU settings [1] [2] [11].

The transition from training to a practicing nurse career has been a difficult problem for the profession to address with very few newly graduated nurses at our institution participating in mentorship program opportunities according to our pre-program survey (Figure 1). The Metro University of Colorado Health Critical Care Mentorship Program was developed to provide structured support to newly graduated ICU nurses during their transition to independent practice. We aimed to develop a program that used a digital platform to facilitate mentor matching, monthly meetings, and structured discussion topics focused on clinical confidence, professional development, transition-to-practice challenges, and emotional support. The program focused on professional support, transition experiences, satisfaction, and retention intent. The aim of this evaluation was to describe mentee perceptions of program benefit and retention intent and to describe mentor-reported satisfaction and retention intent following participation.

## 2. Methods

This post-program observational study evaluated a structured mentorship program implemented across multiple ICUs within a single large academic medical center (University of Colorado Hospital. Anshutz Medical Campus. Aurora, Colorado). Nurses employed in one of 6 ICUs were eligible (Surgical Trauma ICU, Medical ICU, Cardiothoracic ICU, Cardiac ICU, Burn ICU and Neuro ICU). Mentor eligibility included 2+ years of nursing experience. Mentee nurses were matched to an experienced nurse and educator from each ICU. Meetings occurred in person or via Teams during protected time (1 hour per month, paid time for both mentor and mentee) over an exposure period of 6 to 12 months.

Newly graduated registered nurses within their first year of ICU practice were paired with experienced ICU nurse mentors. The program used a structured digital platform to facilitate mentor-mentee matching and ongoing communication. Participants engaged in scheduled monthly meetings over 6 – 12 months, with structured discussion topics addressing clinical confidence, professional development, transition-to-practice challenges, and emotional support. The post program survey was administered after the mentorship period.

Data were collected using separate post-program electronic surveys for mentees and mentors. The surveys included program-evaluation items addressing perceived benefit, job satisfaction, confidence in clinical practice, and intent to remain in ICU nursing, plus open-ended questions regarding program strengths and barriers. The perceived-benefit item used a 5-point response scale (1 = least beneficial; 5 = most beneficial); scores of 4 – 5 were defined as “high perceived benefit,” a score of 3 was “beneficial,” a score of 2 was “neutral or less beneficial”, and 1 was considered “not beneficial”. The mentee and mentor retention-intent questions were worded differently and were analyzed separately.

This research portion evaluating the program with the survey tool was reviewed by the Colorado Multiple Institutional Review Board. Participation in the mentorship program was separate from completion of the evaluation survey. Completion of the survey constituted consent to participate in the evaluation. Survey responses were collected electronically and were anonymous.

## 3. Results

### Participant Outcomes

A pre-program needs assessment survey was conducted amongst nurses that started their careers and currently practice within our hospital ICUs. Prior to the initiation of this program very few new graduate nurses at our institution routinely received mentorship beyond routine training or onboarding (Figure 1). In May 2025, 12 new graduate nurses were hired directly into ICU practice positions at the University of Colorado Hospital. Twelve mentors and the 12 new graduate mentees participated in our newly developed pilot mentorship program. Of the participants, 12 mentors and 11 mentees responded to the post program survey. Participant outcomes are summarized in Table 2. Because the evaluation used a single post-program survey, results are presented as participant-reported perceptions rather than changes from baseline. Mentees described clinical confidence, communication with interdisciplinary teams, and a smoother transition into ICU practice. Five of 11 mentees (45%) selected a high perceived benefit rating of 4 – 5 on the 5-point perceived-benefit scale, while 8 of 11 (73%) selected ratings of 3 – 5. The majority of mentees reported a high level of benefit from the program (Figure 2), citing emotional support, professional guidance, and improved sense of belonging within the ICU team. This has resulted in expansion of the program to areas outside of critical care.

All mentees (11/11, 100%) reported intent to continue practicing as an ICU RN after their first year (Figure 3). Among mentors, 10/12 (83%) reported intent to continue practicing as an ICU RN for the next 1+ years (Figure 4). These intent items were analyzed separately because the questions were used to determine the mentorship program impact on retention of both the mentors and mentees. Eleven of 12 mentors (92%) reported that serving as a mentor increased their satisfaction in their work as a bedside nurse (Figure 5).

Reported barriers included scheduling challenges related to shift work and delayed initiation of mentorship for some participants. Participants described a need for earlier mentorship during the transition from orientation to independent practice. Additionally, participants felt that better matching compatibility (e.g. similar career trajectory, similar interests, etc.) would have enhanced the program outcomes.

## 4. Discussion

This post-program evaluation found that participants reported perceived benefit, satisfaction, and intent to remain in ICU nursing following participation in a structured mentorship program (Figure 6). Among mentees, 5/11 (45%) selected ratings of high perceived benefit and 8/11 (73%) selected beneficial ratings. Additionally, all program mentees reported intent to continue practicing in the ICU after their first year. Among mentors, 11/12 (92%) reported increased satisfaction in their bedside nursing work and 10/12 (83%) reported intent to remain in ICU nursing for the next 1+ years. The high proportion of participants reporting increased satisfaction and intention to remain in ICU practice suggests that mentorship may play a critical role in mitigating early career burnout and workforce attrition. These findings are consistent with existing literature indicating that mentorship enhances professional confidence, supports emotional resilience, and improves retention outcomes among newly graduated nurses [1] [2] [11].

Mentorship likely contributes to burnout mitigation by providing emotional support, fostering professional identity, and reducing feelings of isolation commonly experienced during the transition to practice. The structured and longitudinal design of the program, combined with the use of a digital platform, may have enhanced engagement and accessibility, allowing participants to maintain consistent mentor relationships despite scheduling challenges [1] [2] [11].

Compared to prior studies, the satisfaction outcomes observed in this program are notably high, suggesting that ICU-specific mentorship programs tailored to the unique demands of critical care environments may be particularly effective. Additionally, the strong retention intent observed supports mentorship as a practical workforce stabilization strategy [4] [5] [7]-[10] [12]-[19].

The mentor findings also provide a potentially important perspective. In addition to supporting new graduate nurses, participation as a mentor was associated with self-reported satisfaction among 11/12 mentors (92%). Although this result is descriptive and cannot establish that mentoring caused greater satisfaction, it suggests that future evaluations should consider outcomes for both mentees and experienced nurses who serve as mentors.

From a practice and program perspective, participants’ comments support consideration of initiating mentorship near the end of orientation, protecting time for mentor-mentee meetings despite shift-work schedules, and identifying the staffing and digital-platform resources required to sustain the program. These implementation considerations may be useful to units developing similar programs, but their feasibility and effects on retention require further evaluation.

Several limitations should be considered. The sample was small (11 mentees and 12 mentors), and percentages are therefore heavily impacted by each individual participant. The evaluation used a single post-program survey without a baseline or comparison group, so changes over time and causal effects cannot be determined. Participation may have been influenced by self-selection, and social desirability bias is possible, particularly because the program developer led the initiative. Program exposure varied from 6 to 12 months, and the timing of survey administration relative to program completion varied. The survey instrument was not validated, and the evaluation did not directly measure burnout. The study measured retention intent rather than actual retention. Finally, the program was implemented across multiple ICUs within one institution, and small numbers of participants from each ICU prevent understanding of impact or bias from different ICUs with varying patient populations and workloads. Future evaluations should use validated instruments, standardized exposure periods, anonymous or independently administered surveys, and longitudinal follow-up of actual retention.

## 5. Conclusion

In this small, single-center program evaluation, new graduate ICU nurses and experienced nurse mentors reported perceived benefits associated with participation in a structured mentorship program. Mentees reported strong intent to remain in ICU nursing, while mentors reported high levels of satisfaction and intent to remain in ICU nursing. Larger longitudinal evaluations using validated measures and objective retention outcomes are needed.

## Figures and Tables

**Figure 1.**
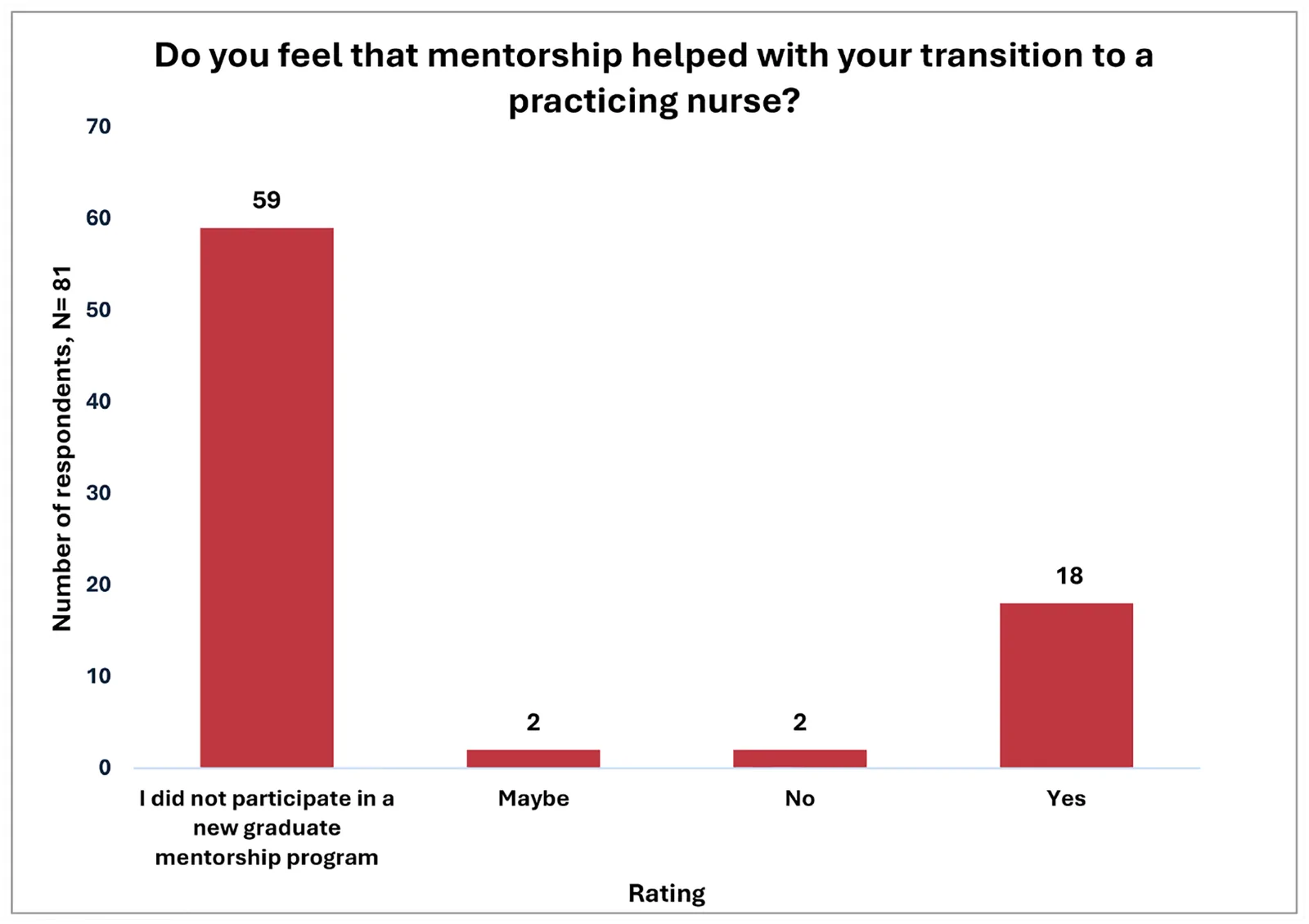
Separate needs-assessment survey of 81 current ICU nurses conducted before mentorship program implementation. Out of 81 respondents, 59 (~73%) did not participate in a mentorship program as new graduate RN. 18 of 22 nurses who stated they had mentorship felt that it helped with transition from new graduate to clinical practice.

**Figure 2.**
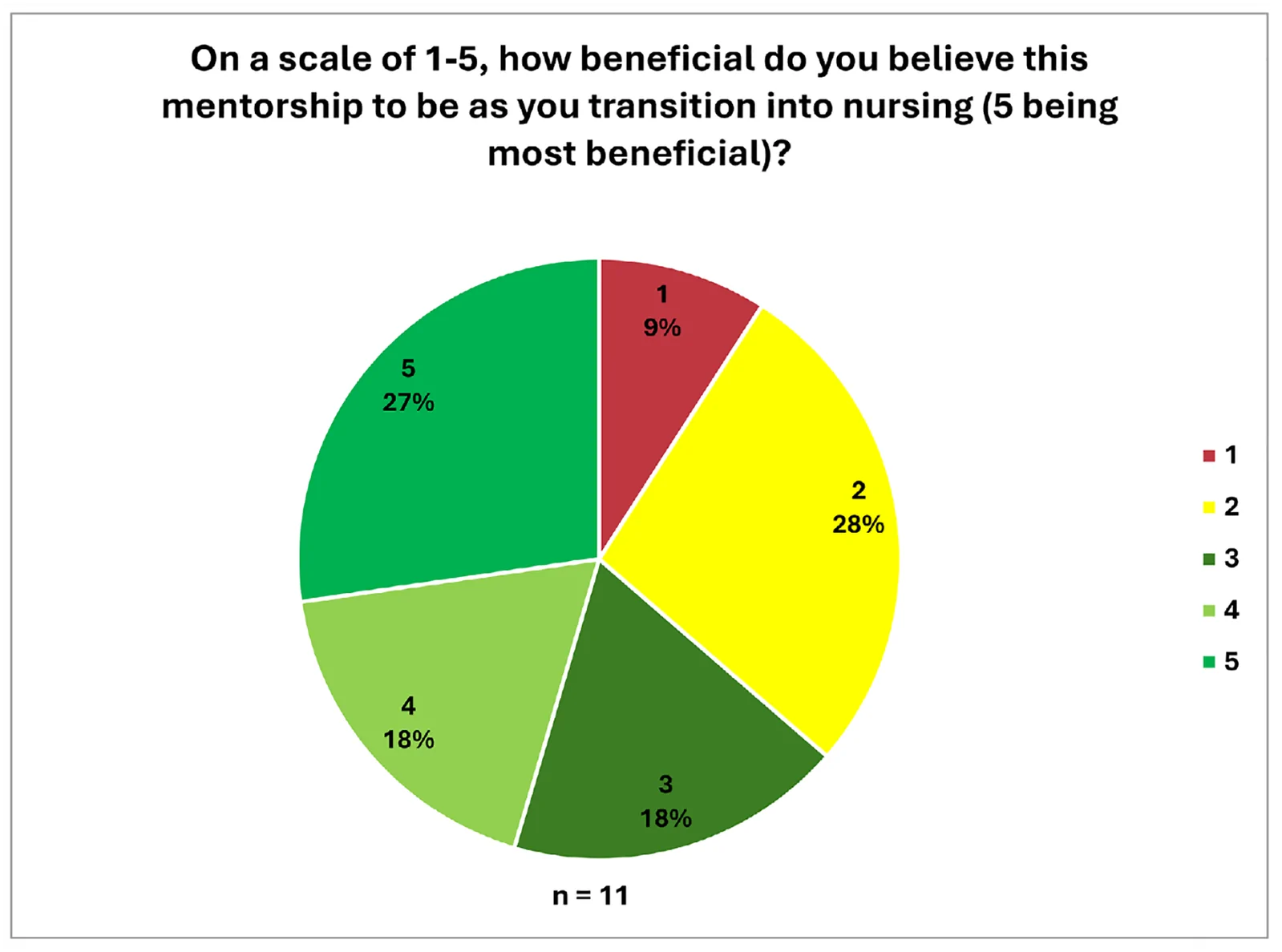
Out of 11 new graduate respondents, 8 (73%) felt that participating in a mentorship program was beneficial to their transition into nursing.

**Figure 3.**
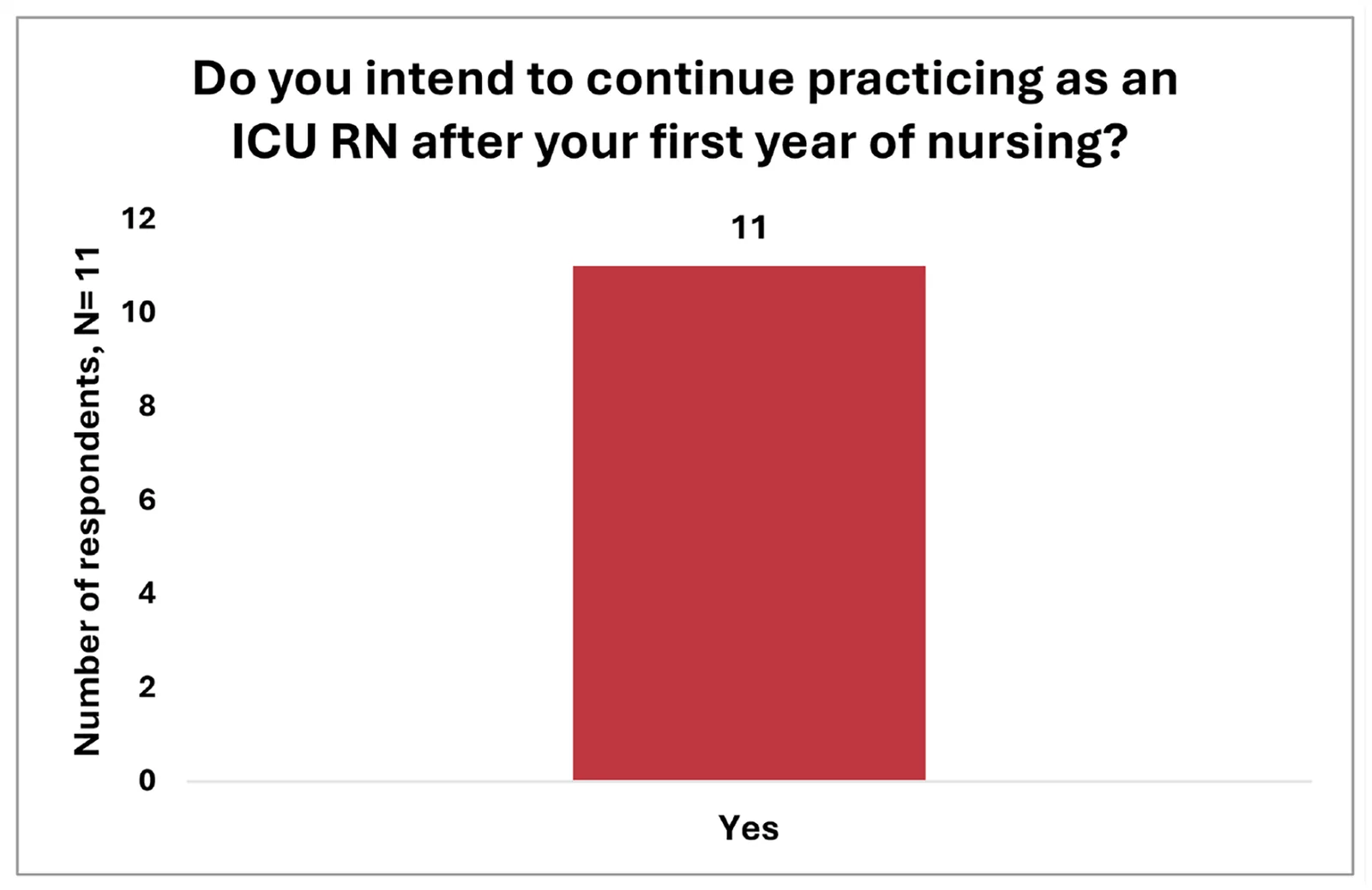
100% of new graduate RNs participating in the mentorship program indicated their intent to remain in ICU nursing after their first year of nursing.

**Figure 4.**
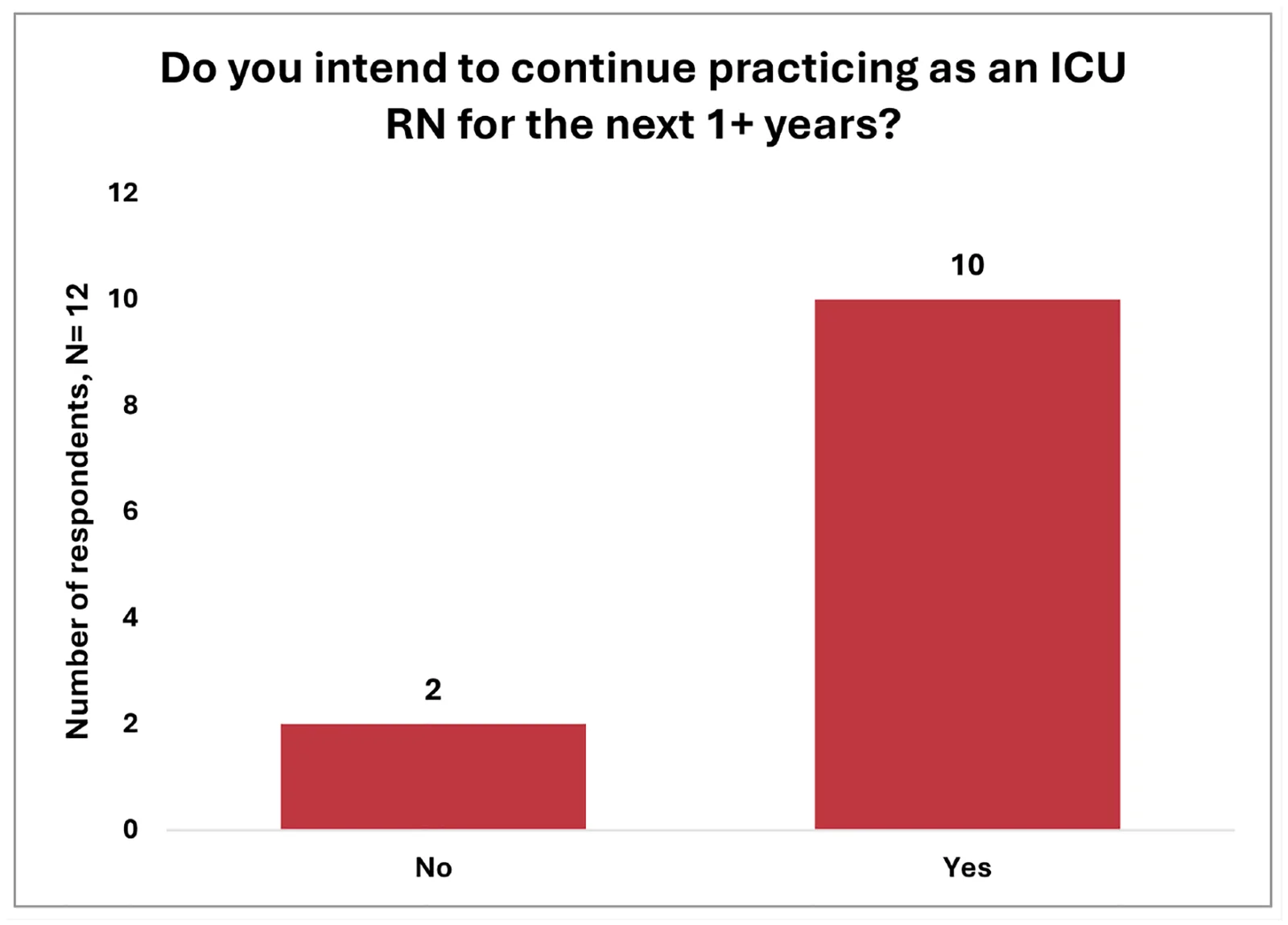
83% of experienced RNs that worked as a mentor indicated their intent to remain in ICU nursing for the next 1+ years.

**Figure 5.**
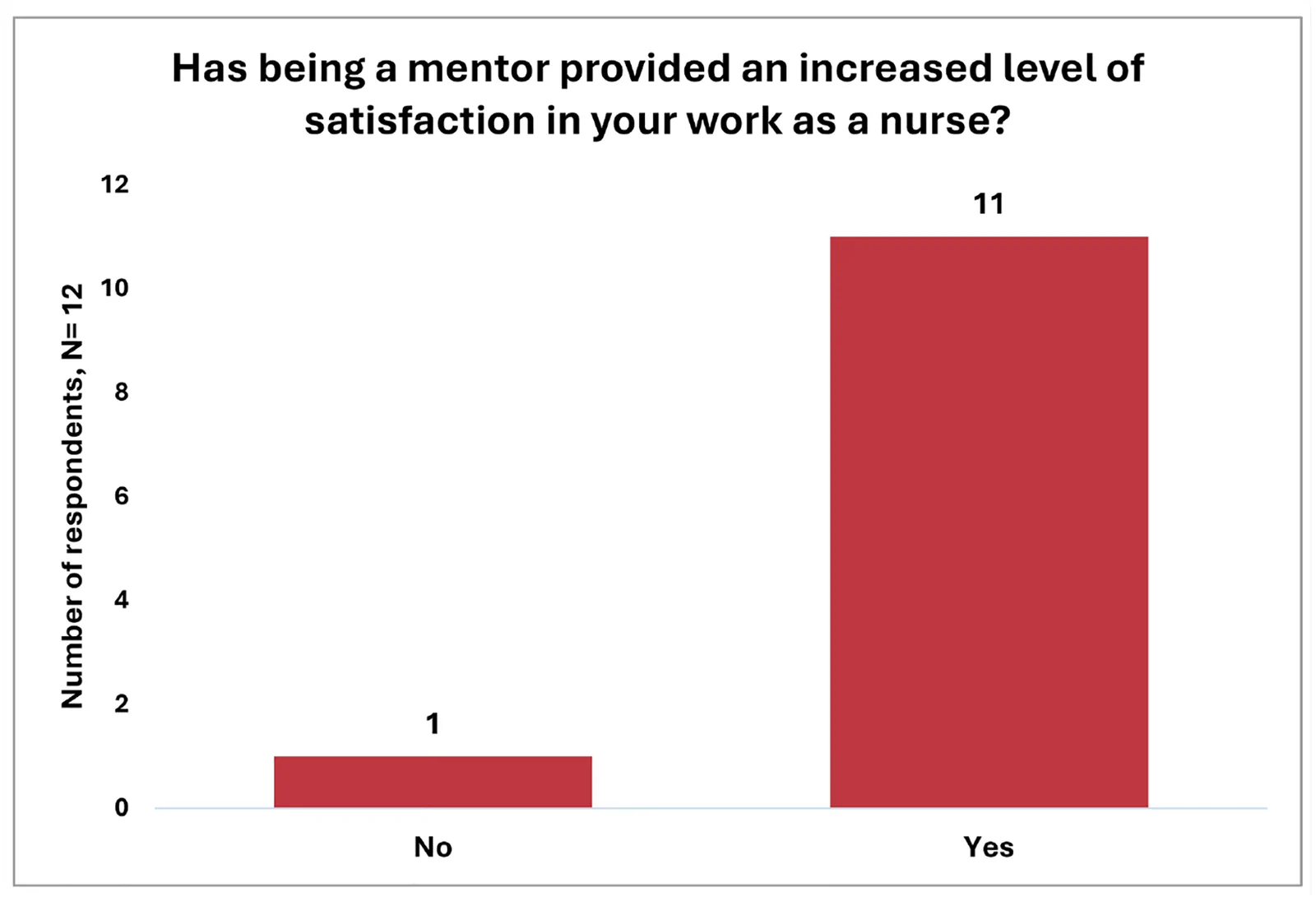
92% of mentors expressed that their work as a mentor enhanced their overall satisfaction in their work as a bedside nurse.

**Figure 6.**
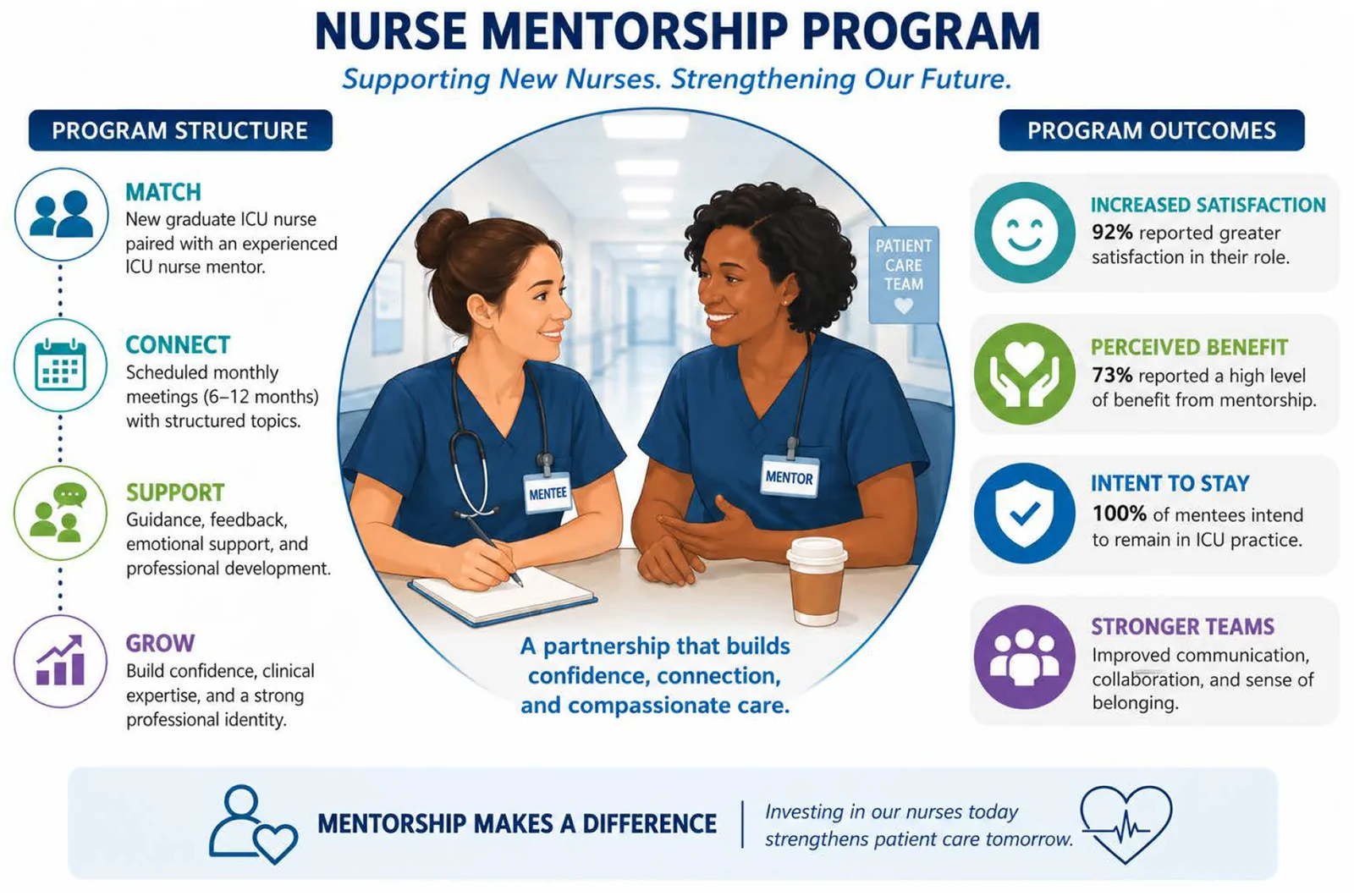
Nurse mentorship structure and programmatic outcomes.

**Table 1.** Summary of literature.

Study	Focus	Findings
Rush *et al*. [11]	Transition programs	Improved competence
Halfer & Graf [15]	Retention	Reduced turnover
Hussein *et al*. [16] [17]	Mentorship	Increased satisfaction
Wei *et al*. [13]	Mentorship	Improved resilience
Brook *et al*. [18]	ICU burnout	Linked to turnover
Figueroa *et al*. [19]	Retention	Mentorship improves retention

**Table 2.** Post-program survey outcomes for mentees and mentors.

Outcome	Mentees, n/N (%)	Mentors, n/N (%)
Perceived high benefit rating 4 – 5	5/11 (45%)	—
Perceived benefit rating 3 – 5	8/11 (73%)	—
Intent to remain in ICU nursing	11/11 (100%)	10/12 (83%)
Increased satisfaction in bedside nursing work	—	11/12 (92%)

Data are presented as percentages.
